# “Just Listen to Me. Help Me Explore it.” An Interpretative Phenomenological Analysis Exploring Experiences of Gender Dysphoria, Identity And Resilience in Autistic Adolescents Assigned Female at Birth

**DOI:** 10.1007/s10803-024-06688-6

**Published:** 2025-01-07

**Authors:** Michelle Oliver, Zoe Poysden, Eimear Crowe, Flo Parkin, Ally Pax Arcari Mair, Neil Hendry, Emma Macey, Karri Gillespie-Smith

**Affiliations:** 1 Department of Clinical Psychology, School of Health in Social Science, Medical School, University of Edinburgh, Teviot Place, Central Campus, Edinburgh, EH8 9AG UK; 2 Psychology Department, NHS Forth Valley, Falkirk Community Hospital, Mayfield Building, Westburn Avenue, Falkirk, FK1 5QE UK; 3 Psychology Department, NHS Lothian, Royal Edinburgh Hospital, Second Floor, Mackinnon House, Morningside Terrace, Edinburgh, EH10 5HF Scotland; 4 NHS Greater Glasgow & Clyde, Young People’s Gender Service, Sandyford, 2-6 Sandyford Place, Glasgow, G3 7NB Scotland; 5 Psych Health, Office 7, 35-37 Ludgate Hill, London, EC4M 7JN UK

**Keywords:** Autism, Adolescent, Gender dysphoria, Qualitative, IPA

## Abstract

Much of the current autism and gender literature has been based within a medical deficiency model; where both are seen as deficiencies rather than differences. However, there is currently minimal knowledge about the experiences of being an autistic adolescent who is both assigned female at birth and has gender dysphoria (GD), whilst even less is known about their experiences of social identity, self-concept and resilience. This study aims to explore experiences of GD with a particular focus on identity and resilience to promote parent and healthcare staff understanding; particularly around gender-affirming care, to foster positive mental health outcomes. Five assigned female at birth autistic adolescents, who identify as trans men/boys, took part in one-to-one semi-structured interviews, which were analysed using an Interpretative Phenomenological Approach. Five subordinate themes arose; *“Having both autism and GD”*,* “Finding where I belong”*,* “Being more than labels”*,* “Trying to cope”* and *“Making support successful”*. Our findings build on current literature and theory regarding the experience of concurrent autism and GD; highlighting its legitimacy, the ways in which autism impacts upon GD and how healthcare approaches and policy can increase accessibility and suitability of gender-affirming care.

**Supplementary Information:**

The online version contains supplementary material available at 10.1007/s10803-024-06688-6.

## Lay Abstract

Gender dysphoria (GD) is a distress people feel when the sex they were assigned at birth does not align with their experienced/expressed gender identity. Very little is known about how this is experienced by autistic individuals, particularly during their adolescent years when socialising and identity exploration are typically key milestones of development. We now know that autistic people labelled female at birth with gender dysphoria display higher levels of suicidality than the general population and non-autistic people with gender dysphoria. As such, we aimed to increase our, parents and healthcare staff’s knowledge of their experiences to better understand how we can help to support these individuals. To do this we interviewed 5 individuals who were autistic, had been labelled at birth as female and had gender dysphoria. We used semi-structured interviews focusing on gender dysphoria, how people felt about their identity and their resilience. We examined the data using an Interpretative Phenomenological Analysis. Five main themes came up; *“Having both autism and GD”*,* “Finding where I belong”*,* “Being more than labels”*,* “Trying to cope”* and *“Making support successful”*. Our findings add to the current literature and theory regarding the experience of autism and gender dysphoria and highlight ways in which healthcare approaches and policy can increase accessibility and suitability of care.

**Supplementary Information:**

The online version contains supplementary material available at 10.1007/s10803-024-06688-6.

The Diagnostic and Statistical Manual (Fifth Edition; DSM-5) describes autism as a condition which includes difficulties with social interactions, social communication and “*restricted*,* repetitive patterns of behaviour*”, which substantially impair functioning (American Psychiatric Association [APA], 2013, p. 31). World prevalence rates have been estimated to be 1 per 100 with 4-4.2 males being diagnosed for every female identified (Baio et al., 2018; Zeidan et al., 2022); although it’s worth noting that there is an issue around the under and mis-diagnosis of autism in those assigned female at birth (i.e., persons whose sex was assigned as female at birth; Hull, & Mandy, 2017).

Gender Dysphoria (GD) is defined as the distress a person could have if their gender identity is not aligned with the sex they were assigned at birth (APA, 2013). Current research posits that autistic people are more likely to experience GD than people in the general population and those with GD are more likely to be autistic than the general population (Glidden et al., 2016; Nabbijohn et al., 2019; van der Miesen et al., 2018a; van der Miesen, de Vries, Miesen et al., 2018a, b; Warrier et al., 2020). This has led some researchers to propose that an association is present; however some of these studies are limited in their methodology; including the use of measures which may not be specific to autism and definitions which may not be specific to GD or trans identity (Thrower et al., 2020; see Turban & van Schalkwyk, 2018, for further detail). The research appears to be set within a medical deficiency model (where both are seen as deficiencies rather than differences), which Shapira and Granek (2019) describe as a cisgenderist-ableist paradigm: where being autistic and/or transgender is seen as being inherently abnormal or lacking.

Despite these controversies within the research field, national guidance in the United Kingdom encourages healthcare clinicians to develop care pathways which meet the needs of all autistic individuals including trans people (National Institute for Health and Care Excellence [NICE], 2012). This is important because research suggests that levels of depression and anxiety are higher in individuals who are trans and autistic than their cisgender autistic and trans neurotypical counterparts (Murphy et al., 2020). What is more, suicide rates have been estimated to be 8.6 times higher in populations with GD (Aitkens et al., 2016). Although there is little evidence of a cumulative risk with each factor, recent findings suggest that numbers of suicide attempts for trans autistic people are higher than trans neurotypicals, with the highest rates being among those assigned female at birth (Polidori et al., 2024; Strauss et al., 2021).

The Minority Stress Model is based upon the premise that those who face discrimination have more stresses, which can lead to poorer health outcomes (Meyer, 2003). This model would suggest that minority stresses can be a risk factor for suicide and mental health problems (Meyer, 2003; Rosbrook & Whittingham, 2010; Winter et al., 2016). Since autistic groups with GD can be seen as a minority within a minority it is ethically essential that more is known about this intersectionality, how mental health outcomes are impacted and how healthcare staff can support autistic people with GD. News like the death of an assigned female at birth autistic transgender man who was reported to have been refused gender-affirming medical support highlights the importance of this (Ellis, 2006). However, the literature is lacking, with experts in the field identifying the need for research into the lived experiences of the autistic trans population to help understand how best to support individuals within healthcare settings (Strang et al., 2020).

Despite the scarcity of research, two qualitative lived experience research studies have been conducted in the adult population. These studies suggest that autism can play both a hindering and/or protective role in GD (Coleman-Smith et al., 2020; Cooper et al., 2022). For example, for some, autism was said to be an obstacle to making sense of their gender-identity, whilst for others it was a factor which supported them to feel more able to live authentically as themselves. These studies also illuminate the experiences of distress from an adult perspective and the barriers to accessing gender-affirming healthcare and mental health support. However, some of the views around the adolescent experiences of GD given could be seen as retrospective as all were adults and many had already accessed gender-affirming medical care (e.g. either hormones and/or surgery).

Further studies have sought views from those earlier on in the experience of having autism and GD (i.e. during adolescence). Two studies investigated the experiences of distress which included the views of 15 young autistic people assigned male or female at birth (Cooper et al., 2022a, 2023). The studies, using the same data, found that young people often looked to medical transition as a way to reduce distress and that adaptation in gender clinics would be required to meet the needs of autistic individuals. Despite being important additions to the knowledge base around distress, a GD diagnosis was not part of the inclusion criteria, limiting homogeneity; creating a need for studies of GD with a more homogenous group.

So far, studies with greater homogeneity in this area have been rare. Strang and colleagues (2018) interviewed 22 autistic trans individuals, assigned male or female at birth with GD between 12.9 and 20.76 years old. They found that participants believed they had difficulties in executive functioning, which impacted their experience of GD and their ability to self-advocate for their own needs. In this study DSM-5 criteria was met for all participants, giving us more confidence that these experiences are those of autistic people with GD.

However, to date, no study has looked at GD solely in young assigned female at birth autistic people. Given that there is a higher risk of suicide for those who are assigned female at birth and there is a potential difference in autistic presentation in those assigned female at birth (including socially gendered expectations), this paper aimed to focus specifically on this group to ensure a more nuanced understanding of their experiences was gained (Murphy et al., 2020; Polidori et al., 2024; Tierney et al., 2016). Furthermore, no study has sought specifically to understand the experiences of group identity, self-concept and resilience; key elements in adolescent development, which may help healthcare staff to have a better understanding of how to support the mental health of assigned female at birth autistic adolescents with GD. As such, this interpretative phenomenological analysis study aimed to explore the lived experiences of GD, group-identity, self-concept and resilience in assigned female at birth autistic adolescents who have not undertaken gender affirming medical interventions (e.g. hormones or surgery). To achieve this, the following research questions were developed;

- What is the experience of gender dysphoria (GD) in assigned female at birth autistic adolescents?
- What are assigned female at birth autistic adolescents with GD’s lived experience of group identity and self-concept?
- How do assigned female at birth autistic adolescents with GD feel about their level of resilience and what supports their resilience?

## Methodological Approach

### Participants

This study recruited 5 assigned female at birth autistic individuals, aged 12–18 years old, who had socially transitioned (i.e. had not received gender-affirming surgery or gender-affirming hormonal interventions) and who were experiencing (at the time of referral into research) GD.

We recruited through both health services and the community. Posters of advertisement were sent to two NHS Scottish health board teams. A total of 5 young people came forward to take part in the study from healthcare settings. Posters were also displayed on social media (namely Facebook, Twitter and Discord) and those interested could contact the researcher directly. One person came forward from the community, however their details could not be verified, and they did not wish to continue.

Participants who took part were on average 16.6 years old (range = 15–18; see Table 1) and had been diagnosed with autism at 13.6 years on average (range = 12–17). Participants all agreed that they have noticed feelings of distress about their gender and on average this was first noticed at 9.8 years (8–14). Participants had all socially transitioned to living as males and had done so on average 4.8 years previously (range = 3–7). All participants identified as either white Scottish or white British. All participants identified as having support from at least some of their family members.

**Table 1:** Participant demographics

Time	Mean (years)	Range (years)
Age at Interview	16.6	15–18
Age at autism diagnosis	13.6	12–17
Age when they noticed distress about their gender	9.8	8–14
Length of time living socially as male	4.8	3–7

Individuals were not eligible to take part in the study if they had been diagnosed with a learning disability, psychosis, bipolar, personality disorder, had transitioned by surgery or had an identified sex development issue (e.g. congenital adrenal hyperplasia or androgen insensitivity syndrome). These exceptions were developed to try to gain a picture of what was more associated with GD and autism rather than other aspects that may influence a person’s experience. In order to ensure safety, those who had been assessed as being “*high risk*” for suicidality (either currently or recently) were also not eligible.

### Procedures

#### Ethical Approval

Ethical approval was given by The West of Scotland Research Ethics Service (WoSRES; study reference number 21/WS/0137). Ethical approval was also sought through the University of Edinburgh. As this study used NHS participants and was across multiple health boards, the Public Benefit Privacy Panel (PBPP), which is concerned with information governance also reviewed and approved the study.

All participants were identified as being eligible by their local healthcare professionals who approached the participant and told them about the study. A referring clinician information form detailed the inclusion/exclusion criteria and steps to take while discussing the research project with a young person. The referrers were able to verify that the individuals had a diagnosis of autism and were experiencing GD as per the DSM-5 criteria.

All participants were deemed by their local healthcare professionals to have capacity to consent. Parental assent was sought from all under 16s with capacity to consent. Those who were interested could give consent to the local healthcare professional to pass on their contact details to the researcher. The main researcher had no prior relationship with any participant and did not know how many participants were approached.

Online semi-structured interviews were offered via Microsoft Teams. Interviews took place between August and October 2022 and lasted 53 min on average, with a range of 40–75 min.

Interview questions (see electronic Supplementary Material 1) were developed using input from clinicians, academics, and an autistic individual with lived experience of GD (lived experienced mentor). A pilot interview with our lived experience mentor helped to refine the interview questions and interviewing approach.

Participants were given the choice to turn their cameras off and type or talk into the microphone. An option to have the interview questions put into the chat was also offered. Directly after the study participants were given a verbal debrief and were sent a copy of this debrief, as well as a support services leaflet for further assistance if they felt any ill effects from the study.

### Data Analysis

All interviews were recorded and for the transcription software on Microsoft Teams was used. The main researcher listened to the recordings to amend and ensure accuracy of the transcription. Video recordings were then destroyed. Once identifying information was removed from the transcripts the data was analysed.

The researcher used Smith and colleague’s six-step process to complete the Interpretive Phenomenological Analysis (Smith et al., 2009). That is, the researcher listened to the audio and read and re-read the transcripts until they felt sufficiently familiar with them. Then, initial noting allowed the researcher to detail descriptive, linguistic and conceptual comments about the texts. From this, emergent themes were developed to capture the essence of the meaning of the researcher’s in-depth coding. Similar themes were clustered into subordinate and superordinate themes and oppositional data was examined.

### Quality Control

In order to reduce bias in the interpretation, Yardley’s (2000) four-part framework (sensitivity and context, commitment and rigor, coherence and transparency and impact and importance) was used to evaluate and promote the quality of this study. The researcher kept a diary to support reflection throughout the process. The main researcher also spoke with participants regarding queried interpretations, to check their validity. A portion of coding was carried out by a second researcher (5–10%) to ensure consistency. Inter-rater reliability was 65%. Themes are the researcher’s interpretations of what the participants’ lived experiences were. To ensure reflexivity, the researcher reflected upon their own social descriptors or Social GGRRAAACCEEESSS (e.g. race, gender and sexuality; Burnham, 2012). Given the writer’s positionality as a non-autistic, cisgender, female adult it was appropriate to include community involvement to ensure sound interpretations of the data, as well as an appropriate and respectfully designed study. Supervision sessions also enabled the researcher to reflect upon this with a fellow researcher.

### Community Involvement

A lived experienced mentor (i.e. an assigned female at birth autistic person who had experienced GD) took part, alongside their parent, in the design of research materials to help ensure that they were appropriate and respectful for participants (including the information sheets, participation poster, debrief forms and support guidance forms). They supported in the review of ethical paperwork, recruitment strategy, analysis of results and dissemination of findings. They also took part in a pilot interview and gave verbal feedback on the appropriateness of the interview and questions. The mentor was given a financial sum as a gesture of thanks for their valued time, funded by the University of Edinburgh. Moreover, a trans autistic researcher provided feedback in the write up of this study. The level of community involvement in this study has enabled us to develop a form of participatory action research which has gained an in-depth and rich perspective on an important topic.

## Results

During the analysis five superordinate themes emerged, each with their own set of subthemes (see Table 2); *“Having both autism and GD”*,* “Finding where I belong”*,* “Being more than labels”*,* “Trying to cope”* and *“Making support successful”.* As recommended by Smith and colleagues (2009), the following results are an analytic commentary of the themes alongside quotations from participants, for clarity and transparency.

**Table 2:** Superordinate themes and subthemes developed using interpretative phenomenological analysis

Superordinate theme	Subtheme	
*Having both autism and GD*	1	“When stuff starts”
	2	Autism’s impact on GD
*Finding where I belong*	1	Difficulty socialising with neurotypicals
	2	The negative impact of transphobia, mis-gendering and bullying
	3	Finding my people
	4	The positive impact of support, validation and acceptance
*Being more than labels*	1	Learning who I am
	2	More than the sum of my parts
*Trying to cope*	1	“I’ve been through hell and out the other side”
	2	Other life difficulties still happen
	3	Strategies to cope
	4	Actively seeking support but in limbo waiting
*Making support successful*	1	Making support successful

### Superordinate Theme 1: Having Both Autism and GD

This superordinate theme highlights the journey that participants have taken to get to where they are now; from a time before dysphoria, to the initial experiences of GD, the way this altered with puberty and coming out socially and the impact that autism has had on the experience of GD.

#### Subtheme 1: “When Stuff Starts”

All participants described that they wanted to be male from a young age;

*“…ever since I was young*,* I wanted to be like a wee boy. I always dressed up as like kinda more like male characters instead of like girl characters…”* (Participant 5).

Some noted feeling like a “*tomboy*” in their early years or being described as such by others, sometimes to their dislike. Many participants were reminiscent about their early years and felt they were largely unaware of the restrictions of the social constructs of gender, with most dressing and engaging in activities that would typically be described as “*masculine*” activities, without feeling a pressure to conform;

*“I don’t feel like I’ve I noticed too much like difference of how I was raised compared to…other guys and stuff…when [we] were like*,* really young*,* we played this thing called Boys Club and it would be like boys versus girls. But I would always be in the boys group… Even though it kind of defeats the whole purpose of the game*,* but I was always set on being in the boy’s group and when I like*,* make believe with my friends and stuff*,* I’d always wanna be a guy character…*” (Participant 4).

Despite feeling that there were not many socially gendered norms put upon him growing up, participant 4 explains in this passage that there were gender labels added in basic play activities by other children and alludes here to the battle beginning by suggesting that he had to advocate for his place in the boys team from an early age. He also implies that he was always striving to be aligned with boys suggesting that the beginnings of identity forming were already at play in a more subconscious way.

All participants described beginning to notice a mismatch between the body and their gender identity between late primary school and early secondary school. This mismatch created distress which was persistent despite participants’ attempts to alleviate it. For one participant this felt like they were disconnected from their own body;

*“Most of the time I don’t feel like my body is connected to me due to it not being the body I feel as though I should have been born with…I think that’s what makes me more distressed about it in general*,* because I look in the mirror and do not see the body I was supposed to be born with*,* you know.”* (Participant 1).

Participant 1 highlights the disconnect between body and mind here, with a body which quite literally did not mirror what he felt he “*should have*”. The use of “*should*” suggests a steadfast resolve to the belief that he was born into the wrong body. The resulting distress was noted by other participants as particularly strong during the start of puberty as the physical changes of the body made the mismatch more prominent;

*“…when stuff starts…when the body started changing like I was quite happy with the way it was. And then obviously it started to develop. So like my chest started to grow…my figure started to kind of change more into like a feminine shape and I hated it*,* I really did. It was horrible.”* (Participant 5).

*“I do remember when I first hit*,* like I got my first period. I felt so disgusted in myself and I didn’t understand why. It wasn’t the blood or anything. It just…people were like ‘ohh*,* you’re becoming a woman now’ or something and it made me uncomfortable. I think it was just the term woman or girl being used for me.”* (Participant 1).

Participant 5’s comment highlights a struggle with change and a pull to reminisce about how things were, in a simpler time before puberty started. His distress is clear within the words “*hate*” and “*horrible*” showing a sense of deep distress with the changes that were happening. This is replicated in participant 1’s comment “*disgusted*”, who also appeared distressed at this change, as unwanted things have happened to him, both on the level of the body change, but also in the way that people started to address him as a woman.

One participant felt that the GD experience was different for each individual. Another felt you didn’t automatically feel like you fitted into the opposite gender;

*“…it’s like not the right fit*,* but not that the other label is necessarily the right fit as well.”* (Participant 3).

These experiences appear to have created a sense of questioning themselves about why they were “*different*”. Some described a feeling that they felt they had to hide their identity to be accepted and actively tried to compensate in order to conform in society, which in turn created more distressing feelings;

*“I wasn’t out at all during high school…”* (Participant 4).

*“I was quite a tomboy for a while like I wore like leather jackets and stuff when I was a little kid. But then I went through a phase of being super*,* super feminine when I first started thinking I was trans; seeing if I could like fit back in*,* like wearing like dresses and stuff. But… it felt like so horrible. Just physically in my body. Like I couldn’t wear those kind of clothes…I just felt like…you know when you’re really cold and you get like*,* all of a sudden*,* a shiver? It was like that kind of underlying feeling just all the time if I’m like wearing like women’s clothing or something*,* like trying to present that way because that’s how it ‘should’.*” (Participant 3).

Participant 3’s extract highlighted the battle back and forth between trying to fit into society and feeling more comfortable within one’s self. Not being true to one’s self appears to have created a visceral reaction, which did not appear to shift.

All participants described the level of distress they experienced; which varied from *“uncomfortable”* to that which caused *“immense mental harm”.* Given that the DSM criteria met highlights GD as “*clinically significant distress*” and this has been assessed by the local healthcare professionals, we may surmise that the purpose of the downplay of distress within such language (i.e. “*uncomfortable*”) may be a coping strategy or linked to a difficulty in language expression (APA, 2013). Four participants acknowledged that they found it difficult to express the level of distress and explain what they experienced given its complexity. Despite not always feeling they had the words to express their pain, all participants gave a sense of this high distress through the repetition of their words;

*“…puberty hit me early*,* unfortunately*,* and it was a bit uncomfortable for me to be*,* you know*,* getting changed…in with all the girls. And they were still in training bras or*,* or hadn’t had puberty yet and*,* and there was me and a full blown bra and it was just uncomfortable. Didn’t like it*,* didn’t know why it was happening. Periods. Oh my God. Hate*,* hate*,* hate it. Every month you had the week of pain and it was just…it was annoying. And I didn’t know why it was happening. And I didn’t like it. And it was just [exasperated noise – ‘eugh’ as exhaling] hate*,* hate it.”* (Participant 2).

Participant 2’s use of the wording “*hit me*” highlighted the sense that this was not something that he was necessarily prepared for and that it had a significant impact on his life. The change appeared to have been both unsettling and physically draining. The repetition of “*hate*” alongside his exasperation goes someway to highlight just how distressing this was for him.

Of note, upon identifying the distress as GD, four participants noted the importance of the correct definition of GD, with some highlighting that it is distress at the “*sex*” assigned at birth rather than gender (as this is not technically assigned at birth). This alludes to a deeper consideration of gender as a social construct.

#### Subtheme 2: Autism’s Impact on GD

This subtheme considers the mixed views around the impact that autism had on experiencing GD. Some participants felt that having both autism and GD made things complex and, at times, it was hard to unpick what influence autism had on GD. One participant questioned whether they would have GD if they did not have autism. They saw gender as a fluid concept and felt a struggle to pick a gender to fit into, but felt they were male because it made the most sense to them;

*“I mean*,* there’s no way to tell at the end of the day*,* like for certain. I can’t say yes or no*,* but if I wasn’t autistic*,* I wouldn’t have um*,* I would have a more structured view of gender. And like for my assumptions like because I don’t have a structure like view of it*,* I’m trans. So maybe if I did I wouldn’t be.”* (Participant 3).

However, others were more certain about their trans identity and there was a sense from most participants that autism helped them; as seeing social constructs in a more critical way helped them to separate their gender identity from their interests;

*“I think it can probably help me separate like kind of separating the gender roles from the actual physical aspects of it. ‘Cause I do have some kind of more masculine interests*,* but I also have plenty of interests that aren’t really masculine. I mean like I like art and I like sewing and stuff like that. And it’s*,* I think it’s kind of helped me separate the*,* the fact that I want my body to be like male doesn’t mean that*,* or isn’t because*,* I have some masculine interests.”* (Participant 4).

Participant 4 highlights that they have spent time considering their GD and have been able to begin to think about gender constructs at a deeper level than might be expected for their age. They showed a separation between masculine activities and masculine identity; highlighting that they can have both alongside things they deem as more feminine activities. The passage implied that autism may support this higher-level thinking. Autism was also seen as helpful to GD in other ways with one participant explaining that his intense interests allowed enjoyable space to be distracted from the distress of GD; supporting his ability to cope.

In terms of sensory difficulties, some participants felt that their adverse reaction to certain textures hindered their ability to “*pass*” as male. For example, the texture of their chest binder was such that they didn’t like to wear it, creating a conflict as they also wanted to wear it to be able to “*pass*” as male in society. One participant felt their aversion to certain textures impacted directly on their ability to dress in dresses and tights, whereas most participants felt that their difficulties with clothing were more about feeling uncomfortable as being perceived as female and had nothing to do with their autism.

Overall, this theme highlighted that participants appeared to have undertaken a long and complex journey starting from a time before socially gendered expectations, progressing to feeling the mismatch in body and mind, which has created distress as well as a sense of needing to hide to conform. The high level of distress has been difficult to express, given its complexity, but is evident in participants’ repetition of their words. This experience was further complicated by having autism, which has been difficult to untangle from the experience. It appeared that autism has had both positive and negative impacts, with various levels of effect on individuals’ GD, which appeared idiosyncratic in nature.

### Superordinate Theme 2: Finding Where I Belong

This superordinate theme reflected the motivations of participants to be social, their difficulties in making friends and their successes. The theme also reflected the experiences and impact of transphobia, bullying and mis-gendering upon them and the positive impact of support, validation and acceptance.

#### Subtheme 1: Difficulty Socialising with Neurotypicals

Some participants described not feeling like they fit in fully with others, that they had tried and failed to fit in at times and they felt a sense of rejection from this;

*“I don’t think I really belong with anyone.”* (Participant 2).

All participants spoke of wanting to socialise with others and had motivation to do so for various reasons. Some enjoyed friendships, some learned from having relationships and others didn’t want to feel like an outcast;

*“I’ve always had friends and I*,* I like talking to other people and learning from having relationships with other people just in different ways.”* (Participant 3).

*“I feel like I don’t fit in with most people*,* but like I kinda like*,* it’s like*,* at one*,* one hand I kinda want to fit in so I don’t look…weird. I…want to like fit so I look normal but then I don’t really want to fit in as well because most of the…the cliques*,* I don’t like them. I’d rather just have my own thing. But being like just if I was to go sit on my own and stuff it*,* I just look at that weird.”* (Participant 5).

Participant 3 appeared to want to socialise because of the benefit of learning he perceived from it. He implied that this may take a slightly different form to what may be expected in a neurotypical social interaction, but that it was okay to be different. Participant 5 alluded to the fact that he naturally wanted to be on his own, but that he did not want to appear different to others; this appeared to be his motivation to socialise.

All participants reflected that they had difficulties with social communication, which they attributed to their autism diagnosis, which made it challenging to socialise;

*“I’ve always found like*,* especially in person*,* social interaction*,* quite difficult I just kind of never know what to say to people I don’t know if I’m talking too much or too little or just kind of gauging conversation in general is really difficult for me. Eye contacts really difficult for me*,* which is really often expected*,* so I’ve always kind of struggled with making friends. The only real way I could do it is if someone approaches me first you know. So yeah*,* I’ve always kind of struggled with that…I think it’s mainly just autism…”* (Participant 4).

However, as noted above, participants’ spoke of hiding their identity in the early stages of realising they were experiencing GD. This could also possibly have impacted upon their ability to socialise, as they were trying to fit into an identity they did not feel comfortable with.

#### Subtheme 2: The Negative Impact of Transphobia, Mis-Gendering and Bullying

Two participants spoke about seeing many instances of transphobia online towards the trans community. Although indirect, this affected participants personally, with one participant feeling that this hate from others had negatively shaped the way he viewed the world. One individual felt able to share his story of directly experiencing transphobia when multiple individuals surrounded him while he was down on the ground. This had and was still having a negative impact on his mental health;

*“…they were all yelling slurs at me and stuff about me being trans*,* consistently misgendering me. And after that I think my dysphoria took a very big toll. I felt so bad. I don’t want to leave the house.”* (Participant 1).

In addition, mis-gendering was also a common occurrence, which had a negative impact upon participants;

*“Well*,* most people they see me and they immediately say she or girl or something. And then when I go “No*,* I’m a guy”. They’ll make excuses for how I’m not a guy sometimes*,* because of my voice or because of the way my hair is or how my body looks and stuff. It’s very damaging*,* but then they’re*,* like*,* show me your birth certificate to prove that you’re not a*,* not a girl. You know*,* that’s happened to me.”* (Participant 1).

Here, participant 1 highlights an experience where others’ made instant assumptions and when corrected doubled-down on their response, making him feel persecuted. It also implied that self-advocating was sometimes not enough to stop others’ negative comments and that the wider-societal response can be harmful.

Other participants also spoke of experiencing feeling bullied, judged, not being believed and experiencing others as not being supportive or accepting of their expressed gender identity. The impact of these types of behaviour from others made participants feel alone and at times abandoned. Some participants noted that they felt bullied by professionals, such as teachers. They felt this was directly linked to their autism and felt it was nothing to do with their gender;

*“She accused me of lying in front of the entire class and kept saying ‘I do not appreciate liars in my classroom. Now sit down’. And I said ‘but I’m not supposed to be here’. She said ‘you can tell me that till you’re blue in the face…You are supposed to be in this class’. She made me cry and I ran away all the way back to the base and phoned my dad. And that woman was horrible.”* (Participant 2).

Overall, these negative instances appeared to be related to both being trans and autistic and there was significant impact on the low mood and anxiety that participants felt; with some having “*meltdowns*” as a consequence of feeling overwhelmed.

#### Subtheme 3: Finding my People

Despite having difficulties with social communication and experiencing transphobia and ableism, participants spoke about finding places that they felt they could fit in, at least to some extent. Two participants noted that they felt they fitted in more with other people who were also either trans and/or neurodivergent;

*“I’ve never really had*,* like*,* specific groups of people that I’m like 100% fit in with*,* but I do get along with multiple different groups of people for different reasons*,* just like if we have similar interests or like*,* I don’t know. I’ve got a couple other trans and autistic friends*,* so I fit in then*,* in that sense.”* (Participant 3).

They described the successes they’d had in meeting new people and forming relationships, both platonic and romantic. This was mostly with people who shared similar interests;

*“Well*,* I mean like my partner right now that I’m going out with right now like we both like music. So like she likes music*,* I like music. That’s kind of one of the things we bonded over at the start.”* (Participant 5).

For some they found they fitted in best in online spaces and for others this was at school or college. One participant felt more comfortable speaking with adults than his peers. Two participants noted that they did not need a large group of people to bond with and were happy with a small friendship circle;

*“I don’t need*,* like*,* a big group of friends. I need about max. 5 friends*,* just people I can do stuff with. That’s all I need…I don’t need to be Mr Popular and have tons of friends if I just have people that I can say*,* ‘hey*,* look*,* you wanna go on a walk*,* like*,* go out and do something?’ or someone I can text at 3:00 o’clock in the morning and have a whine about something then that’s fine…”* (Participant 2).

For Participant 2 “*fitting in*” was not about being the most popular person, or even having a large group of friends, but about having enough friends that he can call on when he wants or needs to engage with them.

#### Subtheme 4: The Positive Impact of Social Transition, Validation and Acceptance

Most participants spoke of the positive impact of coming out to their friends and family as male. One participant felt unwavering in his choice to come out and continued to feel that it was the right choice, which had positively impacted upon his mood. Others felt forced to come out by puberty, but also reported positive experiences directly afterwards, including feeling more comfortable within themselves, building a supportive friendship group and starting to date;

*“But then everyone started to go through puberty and stuff*,* so I kind of had to tell people because it was gonna become really obvious if I didn’t. Um. And that was a little*,* like most people were surprised. A lot of people were confused. Um*,* and it was really good for me as well*,* because everybody who started treating me differently*,* I knew that they weren’t people I wanted to be spending my time with.”* (Participant 3).

*“…we just kept getting kinda closer and then like she told me that she liked me. Then I realized like it was kinda after she told me that she liked me I kinda realized I liked her as well. So then that was like we…started going out after that.”* (Participant 5).

In particular, several participants spoke of the significance of having their hair cut in making them feel more comfortable;

*“I was so happy. Having my first ever short haircut I was ecstatic. Because it felt more like me*.” (Participant 1).

Here participant 1 identified that a physical change, such as a haircut, made an emotional shift towards feeling better about themselves. Overall, there was a sense that getting through puberty and coming out socially changed the experience of dysphoria to one which was more about the mismatch between body and mind than the social aspect;

*“The physical kind of distress about my body itself is still much the same. It’s not as bad as it was*,* but I think that’s just because I’m kind of mostly over puberty now…which is kind of heightens everything*,* but the social distress is a lot better because I’m not getting mis-gendered nearly as often… I wasn’t out at all…so I was constantly kind of getting deadnamed and mis-gendered. But now… I’m out and I don’t deal with any of that. And it’s a lot better.”* (Participant 4).

Participant 4 acknowledged a shift in distress as he came out to friends, but not a total reduction. He implied that after coming out others’ negative reactions towards him lessened. The use of “*deadname*” suggested that by coming out his former identity was well and truly gone, highlighting again the resolve that this participant had about their identity.

All participants acknowledged that they experienced full support from at least one family member, with some having support from both parents and extended family. Two participants acknowledged the journey their families had taken towards acceptance, which was not without difficulty and with resistance at times causing rifts in relationships;

*“…so my dad did pay to get my name legally changed*,* but he was hesitant about it…It’s not that either of them particularly liked my birth name. I don’t think. I think it took them a very long time to actually come to the decision*,* my parents*,* to pick the name in the first place so they weren’t bothered when I changed it. Umm*,* but the pronouns he’s still not quite good at and he says he still sees me as his like little girl*,* which is how it feels*,* which is weird as well because I’m much older. But I don’t really talk to him very often.”* (Participant 3).

*“…they* [his parents] *ended up accepting me. It was a long year. They still messed up*,* but they were getting there and now they have never used my deadname when referring to me for years.”* (Participant 1).

Participants described many parents as being willing learners in their journey to supporting their son which appeared to have a positive impact on both their relationship and their son’s mental health;

*“So I had to basically teach my parents. My father joined groups online to ask questions about trans people. My mum mainly learned from me. She didn’t really mind. I mean*,* she just wanted me to talk to her about it*,* you know*,* and they started actively trying to change their way of viewing things.”* (Participant 1).

It appeared that whilst parental acceptance had a positive impact on the participants, distress was still prevalent, suggesting that more societal change is required to alleviate this distress. Many of the participants acknowledged the need for more acceptance and understanding from wider society. They wanted people to have more understanding about their situation, so that they did not have to explain themselves continually.

Overall, it appeared that socialising could be difficult and at times extremely harmful (e.g. transphobic). Despite this, they had a motivation to socialise, for different reasons and could have successful friendships, particularly when they are related to their intense interests. The support that they had been given from their family and friends, in terms of acceptance and advocacy, appeared to be an important support for all participants.

### Superordinate Theme 3: Being More Than Labels

This superordinate theme highlighted the construction of identity, how the participants saw themselves as more than a dual diagnosis and how they had different experiences of forming a clear identity in adolescence.

#### Subtheme 1: Learning Who I Am

The speed and importance of forming their identity appeared to be mixed for the group. Some felt their identity was very important, whilst for others this appeared to be less important;

*“It’s quite important because like the way that I feel/ identify*,* I want the show that like outwards tae [to] other people.”* (Participant 5).

*“…probably not very [important] seeing as I*,* I don’t really*,* I can’t really like put it in a box.”* (Participant 3).

For some this process was quick and they found their identity was clear from an early age and for others this was an on-going process, which at times still felt unclear. Three participants noted that others had influenced their view on their own identity through their interactions with others;

*“From people like annoying me*,* I’ve kind of discovered that I actually have a really short fuse.”* (Participant 5).

Here, participant 5 implies that he had internalised something about himself being short-tempered because of his interactions with others.

#### Subtheme 2: More Than the Sum of my Parts

Participants acknowledged that both GD and autism were parts of their identity. For different participants each appeared to define them to different degrees, with some being unsure about how much they were defined by their GD and their autism. However, it was clear that most felt that although GD and autism were part of their identity, these parts alone did not define them;

*“I’d say they’re parts of me*,* but I don’t*,* I don’t say they*,* they define that that’s who I am.”* (Participant 3).

Here, participant 3 highlights that he had reached a deeper level in understanding of his identity, tapping into the fact that although his diagnoses were part of him, they didn’t need to be all of him; suggesting his self-concept was well developed.

Instead, the participants identified that there was more to their identity than just their diagnoses; including their interests, values and personalities. Overall, participants highlighted that they were at different stages of their journey in understanding their identity and assigned different levels of value to this; but in the main, most acknowledged that they were more than just their diagnoses.

### Superordinate Theme 4: Trying to Cope

The fourth superordinate theme considered participants’ ability to recognise resilience and explored participants’ feelings of being behind and having other life stressors to deal with on top of those discussed above. The theme also covered the coping skills that these adolescents have picked up along the way whilst having to wait for external supports.

#### Subtheme 1: “I’ve Been Through Hell and out the Other Side”

For some recognising resilience came naturally as they understood the difficulties often associated with transitioning, but could see that they had survived this process and despite this had continued to be themselves;

*“I’m a very resilient person. I’ve*,* you know*,* I’ve been through what I can best describe is*,* I’ve been through hell and out the other side…”* (Participant 2).

Participant 2 was quite aware of the adversities and challenges that life had brought forward for him and he was able to see that this had contributed to the development of his self-concept as a “*resilient person*”, whilst again alluding to having a difficulty with expression.

For others, they felt that others felt they were resilient, but this wavered at times. They could see times where they could show resilience, but at other times they felt this was not the case;

*“It kinda depends on what you say. Like sometimes you can really like touch a nerve. I think everybody’s got that… you could be the most resilient person*,* then somebody really says something that really does touch one of your nerves…Everything else I’m quite resilient*,* but as soon as somebody says something like a certain thing that could like really upset me that really does like knock you back.”* (Participant 5).

Participant 5 alluded to the fact that resilience can be a dynamic process, where resilience can fluctuate. This highlighted his understanding that even the most resilient people can be affected by certain triggers and that he was no different. This speaks to the types of difficulties that he had to face on a regular basis and the development of his understanding of resilience to this level of nuance.

Despite feeling somewhat resilient, three participants noted a feeling of not keeping up and believing they were behind where they wanted to be in life. An indicator for resilience emerged in many interviews that despite the difficulties they faced, participants were able to display empathy towards others who had shown negativity towards them. Some also found hope in optimism and patience.

#### Subtheme 2: Other Life Difficulties Still Happen

Throughout the interviews there was recognition that outside of having GD and autism, there were other things in life which significantly challenged participants; including bereavements and illnesses;

*“I was alone… and my mum wasn’t here. She was looking after my sick [close relative]. So*,* it was just me”* (Participant 2).

*“My [close relative] also passed away a couple years ago*,* so there was that as well.”* (Participant 4).

These difficulties added to feelings of isolation and mental health difficulties, in addition to the difficulties mentioned in previous themes.

#### Subtheme 3: Strategies to Cope

Participants found a range of coping strategies that they described, with some seeming more protective and helpful than others. Some participants felt they just got on with life and tried to ignore their difficulties and keep going;

*“I don’t know how I cope with stuff. I just kind of ignore it.”* (Participant 2).

Several found themselves blaming themselves for the difficulties they faced. Some used distractions, with escapism into online gaming or intense interests. For one individual, trying to cope also led them to feel like they reverted to childhood, by becoming insular and using colouring in books;

*“My brain does this thing that if things get a bit too much*,* my brain like forces itself into…like an age regression…So I can be a bit childish and very quiet and would like to… I don’t know… colour a bloody colouring book*,* that’s for like 3-year-olds. So that’s just how I cope.”* (Participant 2).

#### Subtheme 4: Actively Seeking Support but in Limbo Waiting

All participants described actively seeking support to resolve their difficulties, particularly around the mismatch between the body and the mind and bullying;

*“I think um*,* hopefully I’ll*,* I’ll feel more comfortable in my body and be able to. Um*,* see it a bit more as*,* as me and there’s not like quite a disconnect*,* because I feel like right now there’s a little bit of a… with like there’s me and then my body and there’s*,* they’re not quite together. I’m hoping that will bridge the gap.”*(Participant 3).

Participant 3 spoke here in the context of getting further support from healthcare services and implied a belief or hope that this support would make his GD reduce. “*Bridging the gap*” highlighted a sense that it could be rectified with the appropriate tools and resources. More widely, there appeared to be a quest for acceptance of self, which some participants felt required support from healthcare, family and/or schools to be able to be achieved. Three participants identified that this included medical interventions including testosterone and/or surgery to help resolve their distress.

Despite asking for help, all participants spoke about having long waits for support for GD. For some this wait was currently 2 years, others spoke of waits of up to 5 years. This wait appears to have had a psychological impact upon some participants with them beginning to feel that they might never be helped;

*“It’s just like the feeling of I’m*,* this isn’t gonna happen…if this hasn’t happened for people like me*,* it’s just it’s not gonna happen.”* (Participant 5).

Here participant 5 appeared somewhat hopeless, feeling that support would not come for him. He alluded to a wider issue around intersectionality with his comment “*for people like me*”; suggesting that those in the minority may have a more difficult time accessing services.

Overall, participants have faced significant difficulties related to their autism and GD, but also in areas of life unrelated to autism and/or GD. In response, participants appeared to find ways to cope; some of which were more helpful than others. Some saw the high level of resilience that they had, whilst others did not fully appreciate their resilience level. Participants were actively looking to support their mental health, which was met with a considerable wait for mental health support, or looking for support for their gender dysphoria, which was also met with a considerable wait to attend a gender identity clinic, or both.

### Superordinate Theme 5: Making Support Successful

This superordinate theme considered things participants have found helpful and unhelpful when accessing services regarding their GD and/or mental health difficulties.

Many participants saw the benefit in talking with a professional therapist about their difficulties and some spoke highly of the value and impact that this had/could have had on their mental health. In contrast, for one participant, an initial mental health assessment had felt overwhelming and the approach led to him feeling misunderstood with the result being he no longer felt able to engage with the service; thus the approach created a lack of accessibility for him to mental health care.

There were a number of key things participants identified that made support from professionals successful, including showing kindness, listening empathetically and not jumping to conclusions;

*“That’s what I’d like. Just listen to me. Help me explore it. Come to a solution. I don’t need you to sit there listening to me and then say*,* well*,* it must be this if I’ve already ruled that out.”* (Participant 2).

Here, participant 2 alluded to the fact that there may have been quite a straightforward answer to how to help him feel that he could access support; basic gender-affirming and therapeutically-informed practice.

Participants also spoke of wanting to feel heard by someone who was friendly and caring and who did not pity them. It became clear that those who had reached the top of the gender services waitlist found benefit in the relationships they made with their therapists who emulated many of the positive qualities described above. Participants liked having space to talk to someone without judgement and felt this was beneficial to reduce distress and sometimes led to their diagnosis of ASD;

*“It was just she was just really nice to talk to and really helpful with working through*,

*kind of*,* everything that went on and stuff*,* and she’s actually the one who put me forward for an ASD assessment.”* (Participant 4).

This did not change the way they felt about their gender; instead, the gender-affirming approach helped them to feel more positive about services and have a less distressed relationship with their gender. When support was present it could be overwhelming, but value was given to core gender-affirming and therapeutic skills; including listening empathetically, being accepting and co-constructing a person’s story and goals.

## Discussion

This study interviewed 5 assigned female at birth autistic adolescents with GD to explore lived experiences around GD, group identity, self-concept and resilience. The study used lived-experience mentors (e.g. trans autistic individuals) to co-construct an article which highlights the value and importance of conducting research alongside lived experience; in line with the “*Nothing about us*,* without us*” movement (Autistic Self Advocacy Network, 2021). The results were analysed using Interpretative Phenomenological Analysis to generate a deep and meaningful interpretation of key findings, which were *“Having both autism and GD”*,* “Finding where I belong”*,* “Being more than labels”*,* “Trying to cope”* and *“Making support successful”.*

The first theme *“Having both autism and GD”* highlighted that GD is a complicated and distressing experience for assigned female at birth autistic adolescents who battle between trying to conform to social norms and their internal emotions and beliefs about who they really are, which is further complicated by many factors including having autism and struggling to express their distress. It appears that autism has both positive and negative impacts, with various levels of effect on individuals’ GD; which appear idiosyncratic in nature.

In line with Strang and colleagues (2018), participants describe their very early years as one of relative ease where they were allowed to be themselves, with few socially gendered expectations placed upon them. Distress related to gender started to become evident for participants when social restrictions and expectations became more apparent; around late primary school and early secondary (8–14 years). This is in line, but slightly later than, Strang and colleagues (2018), where most noticed their distress between 5 and 11 years.

Throughout our interviews, distress is described with two key elements; a mismatch between body and mind and the social implications of not conforming. A battle appears to emerge between trying to conform and also trying to be true to themselves; with body dissociation occurring. This journey gives credit to Coleman-Smith and colleagues (2020)’s theme *conflict vs. congruence*, where they describe conflicts with the body and also the social and internal conflicts that arise. It also parallels with more recent autistic trans research and the wider trans research; which also highlights the distinction between body conflict and their distress, suggesting there are multiple conflicts within this experience (Cooper et al., 2022; Cooper, Mandy Cooper et al., 2022a, b, 2023; Galupo et al., 2019; Jessen et al., 2021; Pulice-Farrow et al., 2020).

Interestingly, there appears to be a fundamental change in distress when participants transitioned socially, regardless of whether they felt that this was voluntary or forced by puberty; the battle with socially gendered expectations appears to lessen, whilst self and other acceptance starts to emerge. This change does not appear to impact as much upon the mismatch between body and mind/conflict with the body, which is still prevalent. This may further support current theory that social transition may positively impact upon social conflict, but does not fully resolve GD (Coleman-Smith et al., 2020).

Our research found that puberty is a time when body changes appear to intensify the mismatch between body and mind for assigned female at birth autistic adolescents (Coleman-Smith et al., 2020; Cooper et al., 2022; Cooper, Mandy Cooper et al., 2022a, b). A recent systematic review looking at mothers’ perspectives of parenting autistic daughters, highlighted puberty as a challenge for many assigned female at birth adolescents regardless of gender identity; with resistance to menstruation and hygiene practices being a key finding (Oliver et al., 2024). On the surface this may look to be similar experiences, however, this study illuminates that the experience is much more nuanced; with the negative experience of assigned female at birth autistic adolescents with GD being more about being considered “*a woman*” than the resistance to menstruating or the hygiene practices associated.

Previous studies across autistic adolescent and adult populations have also found that participants appear to struggle with the expression of their distress and thus self-advocacy (Cooper et al., 2022; Cooper, Mandy Cooper et al., 2022a, b; Strang et al., 2018). This paper goes further to spotlight repetition in speech as a potential area that highlights the distress that assigned female at birth autistic adolescents are experiencing. This could be used by healthcare staff as an indicator of distress, to prevent individuals from being excluded from services for being unable to fully convey their distress enough to meet intake criteria.

Our study also highlighted several areas where autism may have impacted the GD experience. Sensory issues appeared to have an impact on assigned female at birth adolescent’s ability to wear a chest binder and “*pass*” as male. This is in line with previous research that highlighted that sensory issues may contribute to GD (Cooper et al., 2022; Cooper et al., 2023). In contrast to others, our study found that intense interests were seen as positively impacting upon GD at times rather than having no impact at all; by being a source of distraction from the GD itself. Autism was also seen as enabling gender to be thought of in a more critical way. In the definition of Gender Dysphoria, the term assigned “*sex*” was preferred by participants over assigned “*gender*” at birth by participants. This demonstrates a deeper level of thinking around social constructs and possibly the performativity of gender (Butler, 1988). Interestingly the International Classification of Diseases 11th Revision (ICD-11; World Health Organisation, 2020) makes this distinction between “*sex*” and “*gender*” in its definition of gender incongruence, whereas the DSM-5 does not (APA, 2013).

This study also highlights that autism adds complexity with competing needs to the GD experience in areas of sensory sensitivities, intense interests and critical thinking; some of which can actively support coping, whilst others can make GD more challenging (Coleman-Smith et al., 2020; Cooper et al., 2022; Strang et al., 2018). Furthermore, battling socially gendered expectations, alongside menstruation, the complexities brought forth by having autism (e.g. making binding more challenging) and also struggling to find ways to express distress presents a unique set of challenges for assigned female at birth autistic adolescents. These conditions create multiple stresses which could impact negatively upon mental health outcomes (Meyer, 2003). It should also be taken into consideration that given our interviews were not exhaustive there are potentially more risk factors for mental health outcomes for this population.

Our second superordinate theme highlighted that although socialising is difficult, the participants were motivated to socialise and mostly found people who they wanted to build friendships with. Motivation to socialise is a feature of other current studies of assigned female at birth autistic individuals (Anderson et al., 2020). Overall, participants found socialising with other trans or neurodivergent peers most successful, particularly when others had similar interests. They appeared to prefer smaller groups and different participants fitted in best in different places (e.g. some in school, some online).

With more general socialising, participants experienced significant physical and verbal transphobia, bullying and mis-gendering. Research in the adult population also highlights that distress can be due to others not being accepting of neuro- or gender-diversity (Cooper et al., 2022), with instances of transphobia being both physical and verbal (Coleman-Smith et al., 2020). As social interactions can result in extremely negative interactions (e.g. transphobia, bullying) there may be potential for individuals to assume some of this onto their own self-identity (Cooley, 1922). Our research highlights that participants blamed themselves at times and felt they had learned negative things about themselves and their identity through interactions with other people. These experiences could induce stress which impacts poorly on long-term mental health outcomes (e.g. minority stress; Meyer, 2003). Our research goes further to show that this can also happen in online environments; with online environments being a double-edged sword; a sanctuary and a treacherous space at the same time (similar to other research carried out in autistic communities being online; Gillespie-Smith et al., 2021).

Something that is less commonly referred to in the literature is the role of family. In this study, participants felt more able to cope when they had support from family. This aligns with the idea that a strong attachment can impact positively on a child’s development and mental health (Bowlby, 1985). This has been shown to be important for transgender adolescents in the wider literature with lack of family support being related to poorer mental health outcomes (Simons et al., 2014; Westwater et al., 2019). For healthcare staff, this highlights the importance of systemic working and supporting those around the child, to support this journey to acceptance, which can indirectly support the young person (NICE, 2012).

Our third theme *“being more than labels”* highlighted that our participants saw their identity as more than the sum of their parts; that they were able to define themselves more holistically than simply through GD and autism. Evidence in other recent studies also suggests that autistic youth have also sought to identify in a more holistic way (e.g. Cooper et al., 2023). Our research highlighted that people felt they were at different stages of identity formation and assigned different levels of importance to it. This may reflect participants’ developmental stage in adolescence, where group identity is formed and self-concept is early in development (Newman & Newman, 2017). This is in line with research with young people who have traits of GD, which highlights that identity development is not impaired (Karvonen et al., 2022). Studies in the wider trans population show that people can struggle with their identity into their adulthood, which gives credit to the idea that this journey of identity forming is a considerable and complex journey (Cooper et al., 2020).

In *“trying to cope”* it became apparent that participants felt they had different levels of resilience, had been through other life difficulties and were trying to cope. Coping strategies employed were mostly self-developed and at times did not feel sufficiently helpful to the individuals. It made sense that participants felt more support was required. It also highlighted that gender transition was important to participants in order to move forward (Coleman-Smith et al., 2020; Cooper et al., 2022; Cooper et al., 2023; Strang et al., 2018). Participants highlighted the long wait times that they were currently experiencing or had previously experienced for either their mental health, GD or both. Wait times appeared to have had an impact in prolonging psychological distress. This is in line with other studies which draw attention to barriers to accessing healthcare (Cooper et al., 2022; Cooper, Mandy Cooper et al., 2022a, b, 2023). Given that physical intervention has been found to improve quality of life and psychological wellbeing, this wait can be seen as a potential risk factor for continued psychological distress (Nobili et al., 2018).

Our final theme *“Making support successful”* showed that when participants attended healthcare support, it was not always accessible to participants; with experiences of being overwhelmed and not feeling heard. This is extremely important as not accessing care also has associated risks for the trans and gender-nonconforming community; including exposure to trauma, poor mental health outcomes, suicidality and self-treatment (Kimberly et al., 2018). For those who did find healthcare accessible they reported this was due to feeling accepted, empathetically listened to and being facilitated to build their story and goals. These skills did not instantly decrease participants dysphoria but enabled them to feel able to continue accessing the support they required; thus gender-affirming and therapeutically informed care are integral to ensure the best possible mental health outcomes for assigned female at birth autistic adolescents with gender dysphoria.

### Strengths and Limitations

Despite recruitment across the UK community and multiple NHS sites a small number of participants showed interest in taking part and more advertising with national third sector organisations would have been useful. However, this number is in line with similar research and is expected given the specificity of the inclusion criteria. Interpretative phenomenological analysis research can be successfully conducted and is appropriate with smaller numbers if the phenomenon of interest is of rare occurrence and allows for in-depth analysis and appreciation of each case (Pietkiewicz & Smith, 2012). Interestingly all of those who participated in the study identified as male. This may limit scope to generalise to non-binary individuals, but made the views heard from a more homogenous group.

The lead author undertook several measures to ensure quality and rigor in this study (Yardley, 2000); including a reflective journal, multiple coders, transparent methods and a thoughtfully chosen analysis method; to ensure depth and reflexivity. This was done alongside someone with lived experience at every stage of the process, from co-creating to write-up, which supported the research to be valid and appropriate. The impact of such research allows us to build on current theory and healthcare policy, as its focus on lived experience and resilience supports a discourse which highlights perspectives of those who can be marginalised in society (Meyer, 2015); using their voices to ensure care is both gender-affirming and accessible.

### Implications

To recap, the implications of this study are numerous. Ultimately, there are clear areas where healthcare can support to reduce stress, which, according to the Minority Stress Model (Meyer, 2003) could lead to better health outcomes. These include practical changes which can be made at health care policy level; including offering quicker support to those waiting with GD and developing familial systems’ understanding of GD in order to support the adolescent. In addition, there is potentially a role within occupational therapy to consider whether there are different usable materials for binders, which could be more suitable for assigned female at birth autistic adolescents with GD. Furthermore, continued professional development around gender-affirming care and how attending to repetition of the words used during assessments may increase the likelihood that young people will feel accepted, empathetically listened to, supported and not missed; making care more accessible.

The study also highlights that there is a wider level of societal responsibility around how we act and treat other human beings, in order to lessen minority stresses (Meyer, 2003). This extends to future research, which has an ethical responsibility to consider intersectionality and how we address certain participant groups and issues. This may allow for research to be conducted giving other groups a suitable voice, for example assigned female at birth autistic adolescents who identify as non-binary. As described above, shifting the language in major texts (e.g. the DSM-5), may support this transition.

## Conclusion

In conclusion, this study explores the unique phenomenological experiences of assigned female at birth autistic adolescents who are experiencing GD. In summary, this includes dealing with socially gendered expectations and internal conflicts, whilst trying to manage menstruation and sensory sensitivities (e.g. which may make binding more challenging), struggling to express this distress, asking for help and showing resilience by trying to cope, but having to wait a considerable time for support. This is on top of experiencing transphobia, mis-gendering, bullying and other life difficulties, such as bereavements.

This paper highlights this as a much-needed conversation and contributes to the growing knowledge about how healthcare staff can support the mental health outcomes of assigned female at birth autistic adolescents. This includes the importance of reducing waiting times, considering the meaning of repetition and utilising key therapeutic and gender-affirming strategies such as acceptance, empathetically listening and co-constructing an individual’s story and goals. In addition, it contributes to recent theory developed to explain experiences within this population (e.g. Coleman-Smith et al., 2020). This paper also shines a light on areas of social and individual identity forming in assigned female at birth autistic adolescents, as well as levels of resilience, validation and acceptance; which can positively impact upon mental health outcomes. Proceeding studies should take stock of the current literature to highlight future areas to explore. Future studies should also give a voice to those assigned female at birth autistic adolescents who identify as non-binary, as well as quantifying the impact of waiting times upon psychological wellbeing - and given current demands on services - striving to find out what waiting “*well*” could look like.

## Electronic supplementary material

Below is the link to the electronic supplementary material.

Supplementary Material 1
