# Supplementary material for: “Just Listen to Me. Help Me Explore it.” An Interpretative Phenomenological Analysis Exploring Experiences of Gender Dysphoria, Identity And Resilience in Autistic Adolescents Assigned Female at Birth
Source: J Autism Dev Disord. 2025 Jan 7;56(5):1934–51. doi: 10.1007/s10803-024-06688-6 (PMC13190371; doi:10.1007/s10803-024-06688-6)
Supplement: Supplementary file 1 — Supplementary Material 1 [file 10803_2024_6688_MOESM1_ESM.docx]

**"Just listen to me. Help me explore it." An interpretative phenomenological analysis exploring experiences of gender dysphoria, identity and resilience in autistic adolescents assigned female at birth.**

Journal of Autism and Developmental Disorders

*Interview Questions and Steers*

| Question Number | Question |
| --- | --- |
| 1 | Being “assigned female at birth” means to be given the gender label “female” on your birth certificate because of your anatomy at birth. What has being “assigned female at birth” (or AFAB) been like for you? Please describe what you have experienced.  *Steer:* How would you describe your gender now? Is this different to the sex that you were born? Tell me more about what this is like for you now and when you were younger. |
| 2 | Are there any ways in which your autism impacts on the way you think or feel about your gender? If so, what are they?  *Steer:* When thinking about your gender dysphoria, are there any things that your autism helps you with? If so, what are they?  *Steer*: When thinking about your gender dysphoria, are there any things that your autism makes more difficult? If so, what are they? |
| 3 | How do you find connecting with other people (e.g. family, friends, and your community)?  Is this affected by your autism, the way you feel about your gender, both or none?  *Steer:* Is connecting with other people important to you?  *Steer:* Do you “fit in” with any group(s) of people? If so, which group and what helps you to “fit in”? |
| 4 | Identity is the idea of knowing who you are as a person. Some people find it hard to know their own identity and others might have a very strong sense of identity.  Do you think you know who you are as an individual? Do you think you know your identity? In what way does you autism and/or gender dysphoria define who you are (or not)?  *Steer:* How important is your identity to you? |
| 5 | Have you learned anything about yourself from other people’s views or the way they act towards you? If so, what? |
| 6 | Resilience is the ability to cope and recover after something difficult happens. How would you describe your level of resilience and what helps you cope?  *Steer:* What strengths do you and/or others who support you have to help you cope?  *Steer:* What would you like to have to help you to cope that you don’t currently have? |
| 7 | Is there anything you could suggest that could help you? If so, what would that be?  *Steer:* Are there any people, groups, activities or services that you have found useful, that you could recommend?  *Steer:* Are there any things that you do (to help your wellbeing) that others could also benefit from? |
| 8 | What else would you like to say that could help others to understand what it’s like to be a person with autism and gender dysphoria? |
| 9 | How has being asked these questions by a researcher felt? Is there anything that could make it better? |
